# Evaluating how lodging affects maize yield estimation based on UAV observations

**DOI:** 10.3389/fpls.2022.979103

**Published:** 2023-01-17

**Authors:** Yuan Liu, Chenwei Nie, Zhen Zhang, ZiXu Wang, Bo Ming, Jun Xue, Hongye Yang, Honggen Xu, Lin Meng, Ningbo Cui, Wenbin Wu, Xiuliang Jin

**Affiliations:** ^1^ School of Geomatics, Anhui University of Science and Technology, Huainan, China; ^2^ Institute of Crop Sciences, Chinese Academy of Agricultural Sciences/Key Laboratory of Crop Physiology and Ecology, Ministry of Agriculture, Beijing, China; ^3^ National Nanfan Research Institute (Sanya), Chinese Academy of Agricultural Sciences, Sanya, China; ^4^ Engineering Mapping and Geographical Information Division, Henan Provincial Communications Planning & Design Institute Co., LTD, Zhengzhou, China; ^5^ State Key Laboratory of Hydraulics and Mountain River Engineering & College of Water Resource and Hydropower, Sichuan University, Chengdu, China; ^6^ Institute of Agricultural Resources and Regional Planning, Chinese Academy of Agricultural Sciences, Beijing, China

**Keywords:** remote sensing, maize yield, lodging levels, random forest regression, UAV images

## Abstract

Timely and accurate pre-harvest estimates of maize yield are vital for agricultural management. Although many remote sensing approaches have been developed to estimate maize yields, few have been tested under lodging conditions. Thus, the feasibility of existing approaches under lodging conditions and the influence of lodging on maize yield estimates both remain unclear. To address this situation, this study develops a lodging index to quantify the degree of lodging. The index is based on RGB and multispectral images obtained from a low-altitude unmanned aerial vehicle and proves to be an important predictor variable in a random forest regression (RFR) model for accurately estimating maize yield after lodging. The results show that (1) the lodging index accurately describes the degree of lodging of each maize plot, (2) the yield-estimation model that incorporates the lodging index provides slightly more accurate yield estimates than without the lodging index at three important growth stages of maize (tasseling, milking, denting), and (3) the RFR model with lodging index applied at the denting (R5) stage yields the best performance of the three growth stages, with R^2^ = 0.859, a root mean square error (RMSE) of 1086.412 kg/ha, and a relative RMSE of 13.1%. This study thus provides valuable insight into the precise estimation of crop yield and demonstra\tes that incorporating a lodging stress-related variable into the model leads to accurate and robust estimates of crop grain yield.

## 1 Introduction

Given the population growth, the demand for food supplies is increasing all over the world (Mishra et al., 2021). Furthermore, limited arable land and frequent extreme weather events have resulted in significant stress on food security. Maize, one of the most important grain crops in the world, is a staple grain crop in China (Jin et al., 2020), where 273 million tons were harvested in 2021, accounting for 40% of global grain production. Timely, accurate, and nondestructive pre-harvest estimation of maize yield is vital for the authorities to formulate corresponding regulation policies and ensure the stability of grain prices and food security (Yu et al., 2020; Feng et al., 2021; Wu et al., 2021). Such estimates also facilitate the development of precision agriculture.

Remote sensing technology can quickly and accurately obtain wall-to-wall information on the land surface and has been widely used to estimate the yield of various food crops (Ju et al., 2021; Maimaitijiang et al., 2021; Nagy et al., 2021). However, satellite remote sensing is often limited by low spatial resolution, long revisit period, and cloudy weather, which prevents image acquisition at certain time points (Xu et al., 2021a). The development of unmanned aerial vehicles (UAVs) provides a way to solve these problems (Peng et al., 2021). As a new tool of information acquisition, UAV remote sensing has irreplaceable advantages in agricultural production (Duan et al., 2021), achieving accurate results with low cost, convenient operation, high spatial-temporal resolution and more (Tao et al., 2020; Wang et al., 2021).

Two main methods exist for crop yield estimation based on UAV remote sensing: data-assimilation methods and statistical models. Data assimilation methods involve crop growth models, which simulate crop growth, development, and yield by combining crop and environment parameters, such as crop species, soil-plant dynamics, water status, and meteorological data (Palosuo et al., 2011; Wu et al., 2021). Assimilating remote sensing data with crop growth models, including WOFOST (van Diepen et al., 1989), AquaCrop (Steduto et al., 2009), DSSAT (Jones et al., 2003), and SWAP (Van Dam et al., 1997), have achieved good results in crop yield estimation. However, these models require a set of biotic and abiotic parameters for model calibration (Kang and Özdoğan, 2019), which undoubtedly increases the complexity of the model because some of the parameters are difficult to obtain. In addition, a certain amount of error exists in most of the environment data acquired from remote sensing data. Consequently, the applicability of the data-assimilation methods is limited in large-scale-yield modeling (Zhang et al., 2019). Statistical models are the earliest and simplest methods to estimate crop yield and are favored by numerous researchers. The basis of statistical models is to establish a linear or nonlinear regression between remote sensing data and measured crop yield (Duan et al., 2017; Zhou et al., 2017). Crop yield estimated by this approach does not address the physiological mechanisms that determine plant growth (Crane-Droesch, 2018), so fewer auxiliary measurements are required. Statistical models to estimate crop yield can be further divided into two categories: linear and nonlinear models. The linear models directly construct the relationship between vegetation indices and crop yield based on linear regressions. However, empirical relationships between crop yield and estimators (e.g., vegetation indices, canopy height, and canopy coverage) usually present nonlinearities (Johnson et al., 2016). Given the generally high degree of autocorrelation of these estimators, yield-estimation models using these variables over time are prone to overfitting. In view of these limitations, machine-learning algorithms were developed to better deal with nonlinearities and reduce overfitting, which is the second type of statistical model. The most successful machine-learning methods for yield estimation include random forest regression (RFR) (Wan et al., 2020; Li et al., 2021), support vector regression (Shafiee et al., 2021), and partial least squares regression (Rischbeck et al., 2016).

Estimating crop yield based on statistical models usually uses spectral, structural, and textural information. Spectral vegetation indices derived from multispectral or hyperspectral data are closely related to some vegetation parameters, such as leaf area index (Tan et al., 2020), green biomass (Li et al., 2020), and crop yield (Panek and Gozdowski, 2021). They describe the average tonal variations in various bands. Structural information such as canopy height and canopy coverage have been used to depict the physiological and geometric characteristics of vegetation and are good indicators of plant growth and crop yield (Malambo et al., 2018). Texture information, characterized by the spatial distribution of tonal variations within a band (Haralick et al., 1973), highlights the structural and geometric features of the plant canopy. Numerous previous studies have estimated crop biomass and yield based on texture (Zheng et al., 2018; Yue et al., 2019; Maimaitijiang et al., 2020). Most studies have focused on using these three common predictors for maize-yield estimates (Rischbeck et al., 2016; Wan et al., 2020). However, few studies have considered how lodging affects crop yield estimation, despite lodging being a common occurrence during the growing season. When lodging occurs, the photosynthetic capacity and dry matter production capacity decrease (Luo et al., 2022), and the transport of water, nutrients, and carbohydrates through the xylem and phloem is cut off (Kashiwagi et al., 2015). During the 12-leaf stage, stalk and root lodging can reduce maize yield by 14% and 28%, respectively (Xue et al., 2017). Every 1% increase in lodging reduces maize yield by an average of 108 kg/ha (Liu et al., 2021). As a result, lodging leads to the loss of crop yield and the reduction of grain quality (Tan et al., 2021). Therefore, lodging may affect yield estimates. To improve crop yield, studies on maize lodging have investigated the factors that cause or affect lodging and have screened lodging-resistant varieties (Chen et al., 2021; Li et al., 2022). In studies of crop yield estimation using agricultural remote sensing, the crop-yield response to lodging has been widely discussed (Acreche and Slafer, 2011; Mi et al., 2011). However, few investigations have focused on how lodging affects model performance and robustness (Chauhan et al., 2019).

To accurately estimate maize yield after lodging, we develop herein a lodging index to represent the degree of lodging of each plot. In addition, we propose a method to estimate maize yield based on spectral features, structural features, texture features, and the lodging index extracted from UAV-based RGB and multispectral images. The analyses were conducted at three different maize growth stages (tasseling stage, milking stage, and denting stage). The specific objectives of this study are (1) to develop an index that represents the degree of maize lodging, (2) to explore how the lodging index correlates with the current model used to estimate maize yield, and (3) to develop a method to accurately estimate maize yield under lodging conditions.

## 2 Materials and methods

### 2.1 Study area and experimental setup

**Figure 1:**
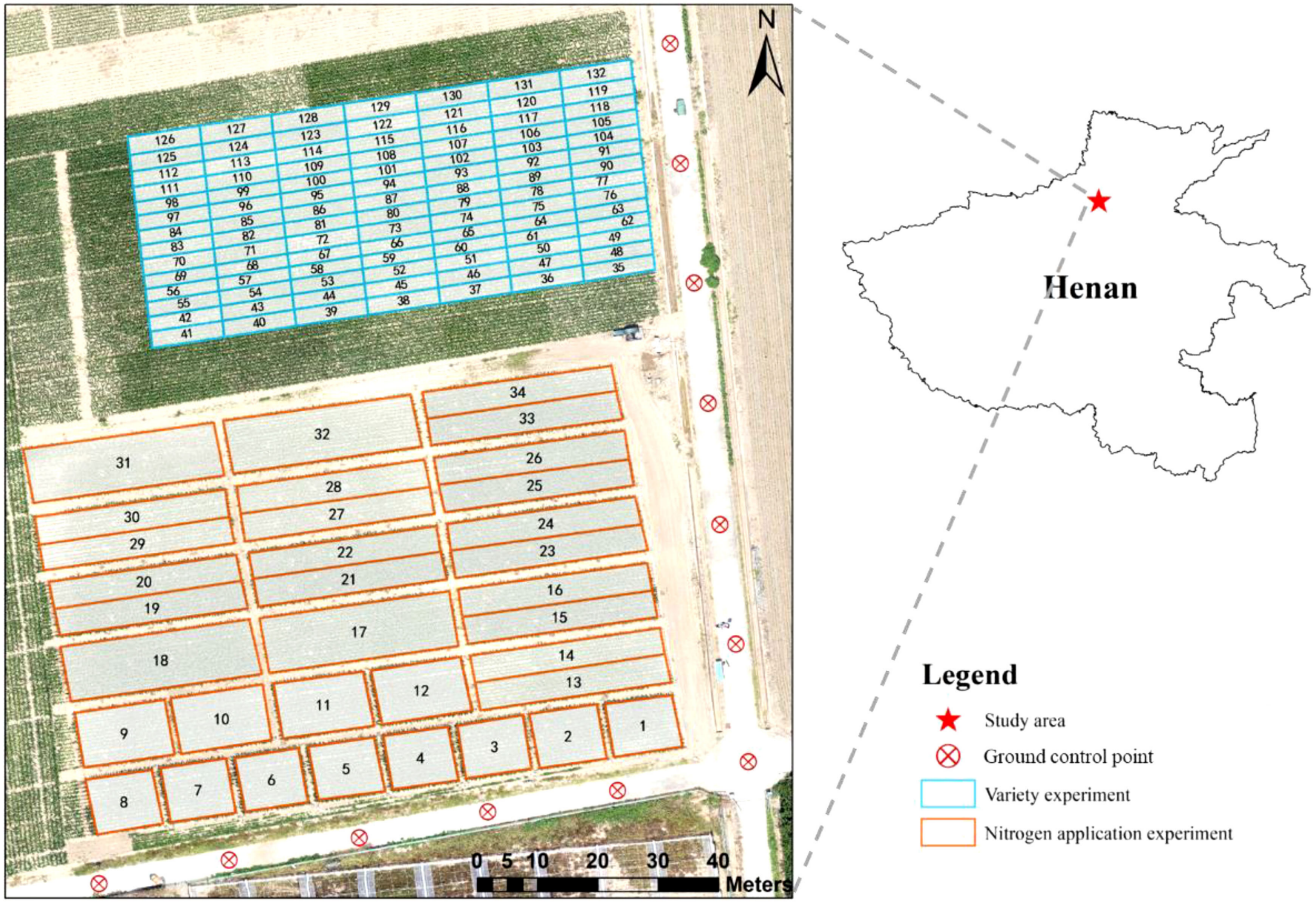
Study area location and layout of the experimental site.

The study area was located at the Xinxiang Experimental Station of the Chinese Academy of Agricultural Sciences (35°7′51.6″N, 113°45′58″E; elevation 75 m), Henan Province in China (**Figure 1**). Xinxiang County is in the North China Plain and has a warm continental monsoon climate characterized by four distinct seasons. The main crop grown in the summer is maize. The average annual precipitation and temperature are 560.6 mm and 14.3 °C, respectively. The average annual precipitation is unevenly distributed, being mostly concentrated between June and September, when about 75% of the annual rainfall occurs.

Maize was planted in 132 plots with different varieties and fertilizer treatments (**Figure 1**) to ensure the generalizability of the proposed method and avoid overfitting. In the variety experiment, 98 maize varieties with different genotypes and lodging resistance were planted, each in a 3 m × 10 m plot. The same fertilizer treatment was applied in each plot. In the nitrogen-treatment experiment, two maize varieties widely planted in north China (JNK728 and ZD958) were planted in 34 plots, with varied fertilizer treatment (0–400 kg/ha) and application time (from before sowing to the silking stage). In all the plots, urea (CH_4_N_2_O) was used as nitrogen source. The planting density was 75 000 plants/ha, with a row spacing of 60 cm, and a plant spacing of 23 cm.

### 2.2 Data acquisition

#### 2.2.1 UAV images acquisition

**Figure 2:**
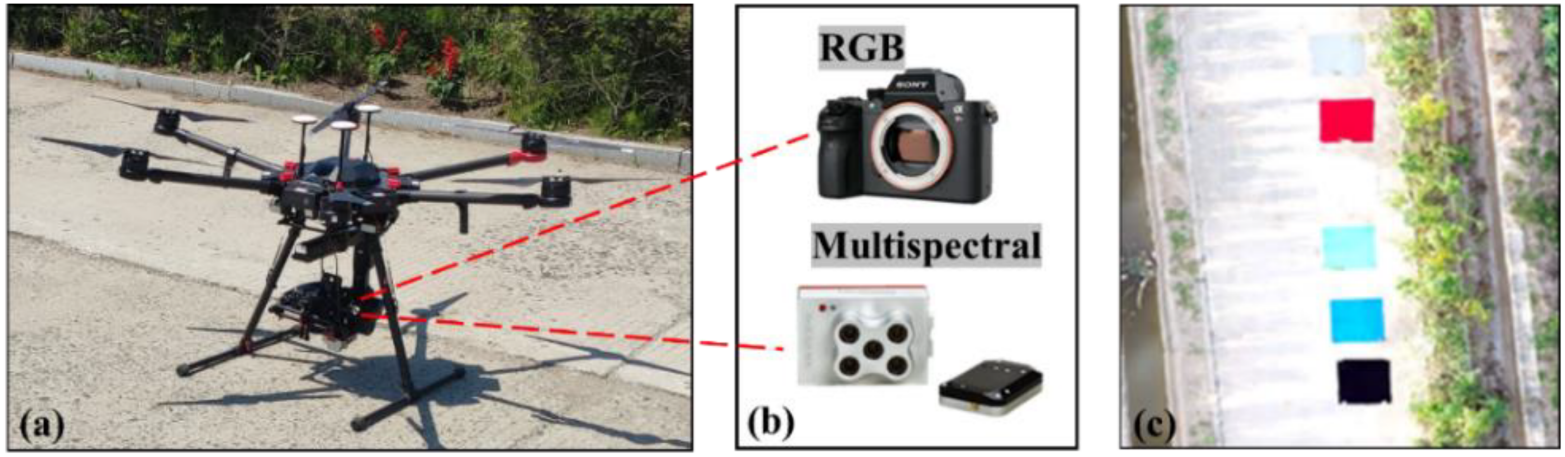
The UAV platform and sensors: **(A)** DJI M600 pro, **(B)** Sony α7RII and MicaSense RedEdge-MX, and **(C)** six tarpaulins of different colors.

This study used a DJI M600 pro UAV (**Figure 2A**, DJI Innovation Co., Ltd., Shenzhen, China) equipped with a Sony α7RII and a RedEdge-MX camera to collect RGB images and multispectral images, respectively (**Figure 2B**). The Sony α7RII digital camera uses a complementary 35.9 × 24.0 mm^2^ metal-oxide semiconductor sensor with a resolution of 42.4 million pixels. The RedEdge-MX multispectral camera includes five bands (blue or B, green or G, red or R, red edge or RE, and near-infrared or NIR) within the spectral region of 400–1000 nm. The details of each sensor are given in **Table 1**.

**Table 1:** Basic parameters of the sensors mounted on the UAV.

Sensor name	Sony α7RII	RedEdge-MX
Sensor type	RGB (Red-Green-Blue)	Multispectral
Dimension (mm)	126.9×95.7×60.3	87×59×45.4
Spectral region (μm)	N/A	Blue:0.475, Green:0.560, Red:0.668, Red-edge:0.717, Near-infrared:0.842
Resolution (pixels)	7952×5304	1280×960
Focal length (mm)	70	5.4
Mass (g)	625	231.9

UAV images of summer maize were collected in 2020 at the stages of tasseling (VT), milking (R3), and denting (R5). Three flight missions were undertaken at 30 m height and a speed of 2.1 m/s. Each flight mission took about 30 minutes. The lateral and forward overlaps were 80% and 90%, respectively. Each flight campaign was conducted during clear and sunny weather between 12 p.m. and 2 p.m. to reduce the impact on image quality of cloud cover and changes in solar zenith angle. To calibrate the multispectral camera, the FieldSpec 4s spectroradiometer (Analytical Spectral Devices, Boulder, Colorado, USA) was used to measure the reflectivity of tarpaulins with six different colors (gray, red, white, green, blue, and black) at various wavelengths in the study area (**Figure 2C**). The spectral range of the spectrometer was from 350 to 2500 nm, each color tarp was measured ten times during the UAV flight and the average was taken as the reflectance result of the corresponding calibration tarp. The six color tarpaulins were placed in the field perpendicular to the flight path, allowing their reflectance data to be acquired during the UAV flight. Meanwhile, to facilitate the georegistration of images, 12 ground control points (GCPs) were evenly marked around the plots (**Figure 1**).

#### 2.2.2 Collection of yield data

**Figure 3:**
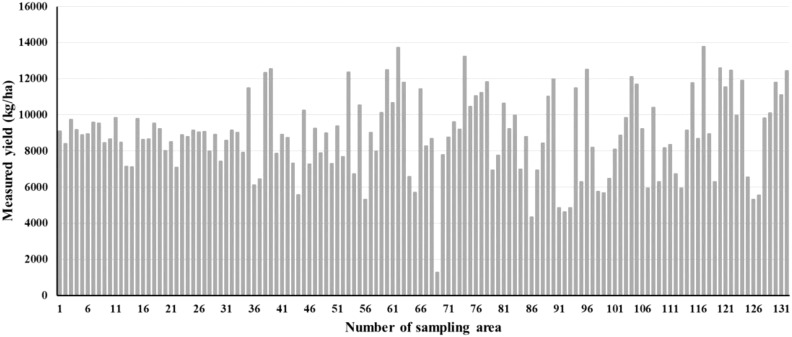
Bar plot showing the measured maize yield in the sampling plots.

The yield of the 132 plots was measured by using the following procedure: For each plot, all maize plants in six 5-m-long rows were harvested. Ten maize ears of representative size and weight were selected for indoor measurement of water content and grain weight. The relative water content of the grain was measured with a PM-8188-A Grain Moisture Meter (Kett Electric Laboratory Co., Ltd., Tokyo, Japan). Finally, the grain yield at 14% water content of each plot was calculated and is shown in **Figure 3**. The lowest yield (1291.08 kg/ha) was from plot 69. The highest yield (13781.02 kg/ha) was from plot 117.

### 2.3 Image preprocessing

The orthoimages of RGB and multispectral data were generated using the Agisoft PhotoScan software (version 1.4.5, Agisoft LLC, St. Petersburg, Russia), facilitated by the GPS and IMU (Inertial Measurement Unit) data recorded by the UAV flight control system. Georegistration of the orthoimages obtained at different times was conducted so that the positional displacements were removed, and the images were geographically well aligned. This step was done by the ArcGIS software (version 10.4, Environmental Systems Research Institute, Inc., Redlands, CA, USA) according to the 12 GCPs (**Figure 1**).

**Figure 4:**
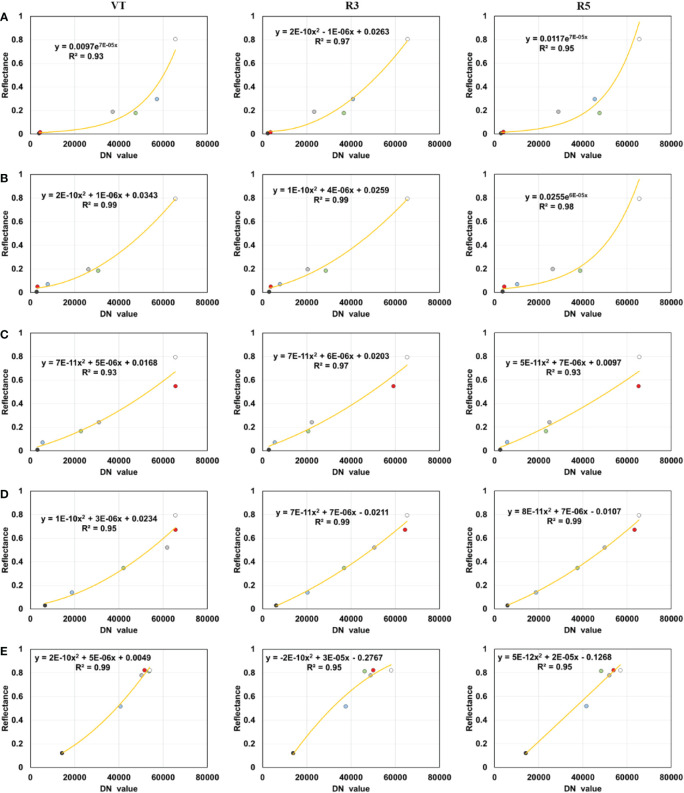
Multispectral radiometric calibration fitting models. **(A)** blue band, **(B)** green band, **(C)** red band, **(D)** red-edge band, and **(E)** near-infrared band.

**Figure 5:**
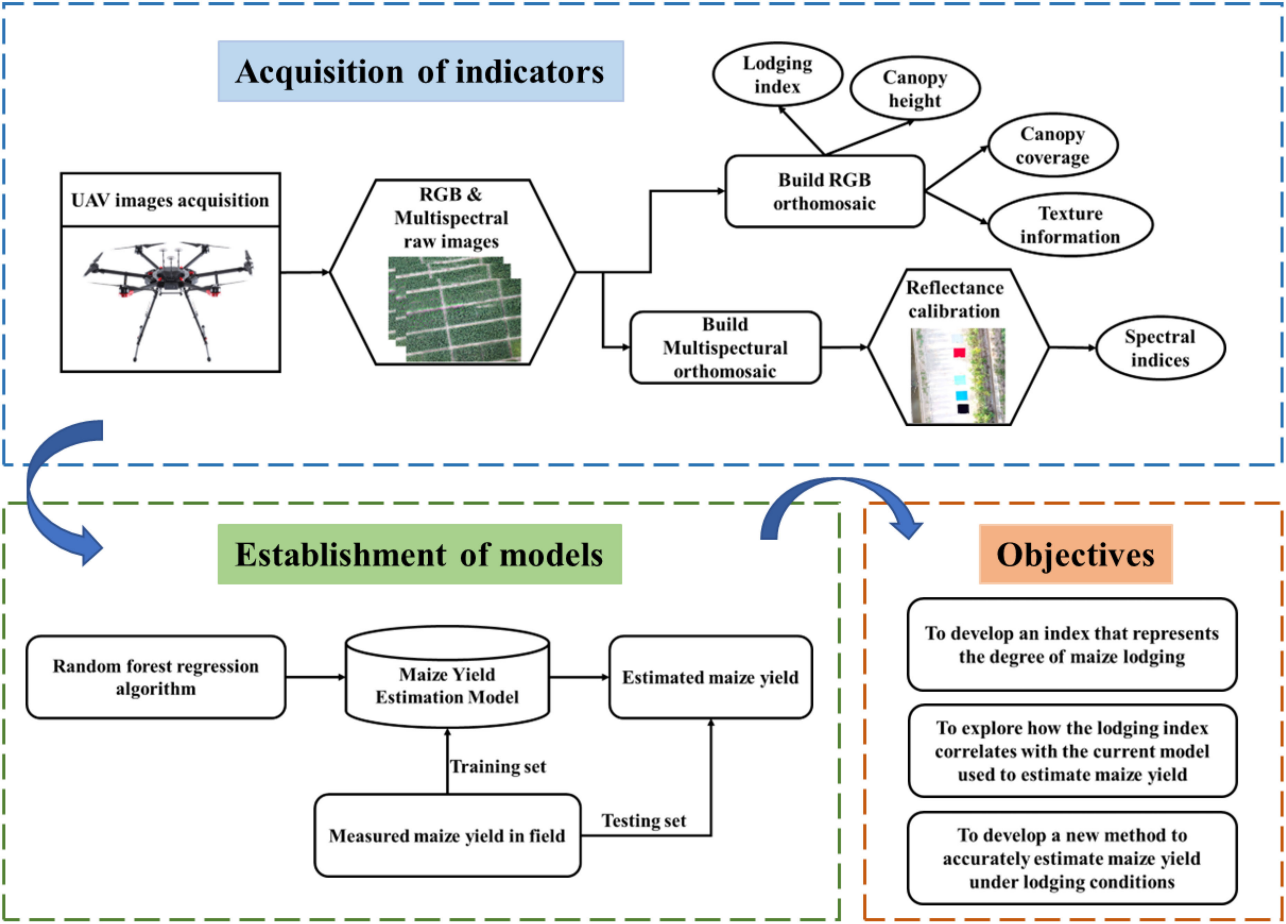
Workflow for preprocessing, feature extraction, model building, and target research using multimodal data.

For the multispectral images, radiometric correction was conducted to convert image digital number (DN) values into reflectance to extract spectral information. Linear, quadratic, exponential, logarithmic, and power functions were used to fit the relationship between the DN values of six color tarpaulins (**Figure 2C**) extracted from the images and their reflectance based on the FieldSpec 4s spectroradiometer measurements. The optimal functions were used to separately calibrate each band in each growth stage (**Figure 4**). The exponential function was selected for the blue band in the VT and R5 stages, and for the green band in the R5 stage, with R^2^ reaching 0.93, 0.95, and 0.98, respectively. The quadratic function, with R^2^ varying from 0.93 to 0.99. The overall workflow of this study is shown in **Figure 5**, including the acquisition of indicators, the establishment of models, and the research objectives.

### 2.4 Features extraction from multimodal images

Integrating multimodal features for crop yield estimation has been validated in previous studies (Rischbeck et al., 2016; Feng et al., 2020; Ramadanningrum et al., 2020). Estimating maize yields by integrating spectral, structural, and textural information can provide more information related to yield and overcome the inherent asymptotic saturation of single canopy features (Maimaitijiang et al., 2017). In this study, the lodging information was also considered.

#### 2.4.1 Canopy spectral information

The spectral characteristics of crops are affected mainly by the absorption, reflection, and transmission of electromagnetic radiation caused by the physiological structure of plants, which makes spectral indices good predictors of crop yield (Berger et al., 2020; Yang et al., 2021). In this study, radiometrically calibrated multispectral images were used to extract the canopy spectral features. Fourteen spectral indices that have been widely used in yield estimation were selected (Sui et al., 2018; Maimaitijiang et al., 2020), as shown in **Table 2**. The extraction was implemented in the ArcGIS software.

**Table 2:** Definitions of the features extracted from multispectral imagery.

Spectral index	Formula	References
Normalized difference vegetation index	*NDVI*=(*NIR*−*R*)/(*NIR*+*R*)	(Rouse et al., 1974)
Ratio vegetation index	*RVI*=*NIR*/*R*	(Tucker, 1979)
Green-red vegetation index	*GRVI*=(*G*−*R*)/(*G*+*R*)	(Tucker, 1979)
Optimized soil adjusted vegetation index	*OSAVI*=(*NIR*−*R*)/(*NIR*−*R*+*L*)(*L*=0.16)	(Rondeaux et al., 1996)
Normalized difference red-edge	*NDRE*=(*NIR*−*RE*)/(*NIR*+*RE*)	(Gitelson and Merzlyak, 1997)
Modified chlorophyll absorption in reflectance index	*MCARI*=[(*RE*−*R*)−0.2∗(*RE*−*G*)]∗(*RE*/*R*)	(Daughtry et al., 2000)
Transformed chlorophyll absorption in reflectance index	*TCARI*=3∗[(*RE*−*R*)−0.2∗(*RE*−*G*)∗(*RE*/*R*)]	(Haboudane et al., 2002)
Green normalized difference vegetation index	*GNDVI*=(*NIR*−*G*)/(*NIR*+*G*)	(Gitelson et al., 2003)
Wide dynamic range vegetation index	*WDRVI*=(*a*∗*NIR*−*R*)/(*a*∗*NIR*+*R*)(*a*=0.12)	(Gitelson, 2004)
Green chlorophyll index	*GCI*=(*NIR*/*G*)−1	(Gitelson et al., 2005)
Red-edge chlorophyll index	*RECI*=(*NIR*/*RE*)−1	(Gitelson et al., 2005)
Two-band enhanced vegetation index	*EVI*2=2.5∗(*NIR*−*R*)/(*NIR*+2.4∗*R*+1)	(Jiang et al., 2008)
Normalized difference red-edge index	*NDREI*=(*RE*−*G*)/(*RE*+*G*)	(Hassan et al., 2018)

#### 2.4.2 Canopy structure information

The canopy-structure information is directly related to light use efficiency (Xu et al., 2021b), which contains independent information from spectral and texture feature (Stanton et al., 2017). Two indicators, canopy coverage (CC) and canopy height (CH), were extracted to characterize the structure of the maize. Both were extracted from the UAV-RGB images.

Canopy height was extracted by generating a crop height model (CHM) from UAV high-resolution RGB images. At each of the three growth stages, the structure-from-motion (SfM) algorithm was implemented in Agisoft PhotoScan software to create a three-dimensional (3D) points cloud and a digital surface model (DSM) (Xie and Yang, 2020). In addition, a bare-earth digital elevation model (DEM) was created based on photogrammetric 3D point clouds generated before maize emergence (Maimaitijiang et al., 2019). Subsequently, the CHM was calculated by subtracting DEM from DSM using the raster calculator tool in the ArcGIS software.

The calculation of CC depends on the correct recognition of crop pixels from among the background (soil and weeds). Previous studies have shown that vegetation and soil can be separated based on indices calculated from RGB images, such as the color index of vegetation extraction (CIVE), excess green index (ExG), and excess green minus excess red (ExG − ExR) (Hamuda et al., 2016; Castillo-Martínez et al., 2020). In this study, the CIVE [Eq. (1)] is used with a threshold interval set at [−28, 5]. The CC is thus expressed as the fraction of maize canopy pixels to all pixels in the sampled plot [Eq. (2)]:


(1) CIVE=0.441R−0.811G+0.385B+18.787

(2) $$CC=\frac{P_{maize}}{P_{total}}\times 100\%$$

where *P_maize_* is the number of maize canopy pixels the plot, and *P_total_* is the total number of pixels in the plot.

#### 2.4.3 Canopy texture information

Canopy texture can provide additional information related to spatial canopy architecture and spectral characteristics (Xu et al., 2022). The inclusion of texture features can reduce the bias of yield estimates beyond what is possible using spectral indices alone; this is helpful for early monitoring of grain yield (Wang et al., 2021). In this study, texture features were extracted from UAV-RGB images based on the gray-level co-occurrence matrix (GLCM), which is a popular method to extract texture features (Mohanaiah et al., 2013). This step was implemented in the ENVI software (version 5.3; Esri Inc.) with a window size of 7 × 7. The meanings and formulas (Haralick et al., 1973) of the texture indices are presented in **Table 3**.

**Table 3:** Meanings and formulas of the selected texture indices based on GLCM.

Texture index	Formula	Meaning
Mean	$\begin{array}{l} \mu_{i}=\sum_{i,j=0}^{N-1}i\left(P_{i,j}\right)\\ \mu_{j}=\sum_{i,j=0}^{N-1}j\left(P_{i,j}\right) \end{array}$	Average gray level in the window.
Variance	$\begin{array}{l} \sigma_{i}=\sum_{i,j=0}^{N-1}P_{i,j}(i-\mu_{i}{)}^{2}\\ \sigma_{j}=\sum_{i,j=0}^{N-1}P_{i,j}(j-\mu_{j}{)}^{2} \end{array}$	Variance of gray level in the window.
Homogeneity	$HOM=\sum_{i,j=0}^{N-1}P_{i,j}/\left[1+{\left(i-j\right)}^{2}\right]$	Measure of the homogeneity across the window.
Contrast	$CON=\sum_{i,j=0}^{N-1}P_{i,j}{\left(i-j\right)}^{2}$	Metric of the local change in pixel value between adjacent pixels.
Dissimilarity	$DIS=\sum_{i,j=0}^{N-1}P_{i,j}\left|i-j\right|$	Metric that reflects the difference in grayscale.
Entropy	$ENT=\sum_{i,j=0}^{N-1}P_{i,j}(-\ln P_{i,j})$	Measure of the disorder across an image.
Angular Second Moment	$ASM=\sum_{i,j=0}^{N-1}P_{i,j}^{2}$	Metric of the uniformity of the image gray level distribution.
Correlation	$COR=\sum_{i,j=0}^{N-1}P_{i,j}\left[\left(i-\mu_{i}\right)\left(j-\mu_{j}\right)/\sqrt{\left(\sigma_{i}^{2}\right)\left(\sigma_{j}^{2}\right)}\right]$	Metric of linearity between adjacent pixels.

From Hall-Beyer (2017); Park and Guldmann (2020), and Haralick et al. (1973).

N is the number of gray levels. i and j are the column and row labels of the GLCM, respectively. P_i,j_ is the probability that values i and j appear in the adjacent pixels of the original image within the window that defines the neighborhood. μ is the mean and σ is the standard deviation, defined by the GLCM mean and the GLCM variance equation in the table.

#### 2.4.4 Lodging stress information

Around August 3, 2020 (tasseling stage), a sudden rainstorm accompanied by strong winds (wind force level 5 to 6) hit the experiment site, resulting in varying degrees of lodging in the maize plots. The distribution and degree of lodging were investigated in the field the day after. Different degrees of lodging occurred in the same plot at the pixel level. In this study, the lodging degree at the pixel level was extracted as per Wang (2021) and then divided into three categories: no lodging (NL), light lodging (LL), and severe lodging (SL). NL means that the maize remained upright, and the angle between the plant and the ground was between 0° and 30°. LL means that the angle between the maize plant and the ground was 30° to 60°. SL means that the maize plant was close to or completely on the ground. An index representing the degree of lodging of each plot was developed based on the pixel-level degree of lodging and the lodging area in each plot. The calculation was implemented in the ArcGIS software. The formula for calculating the lodging index is


(3) $$LI=\sum_{i=1}^{3}LD_{i}\times S_{i}$$

where *LI* is the lodging index of a plot, *LD_i_* is the pixel-level lodging degree, and *S_i_* is the area fraction of lodging degree *i* in the plot.

### 2.5 Random forest regression algorithm

We selected the RFR machine-learning algorithm because it has already produced accurate and robust estimates of crop yield (Aghighi et al., 2018; Cai et al., 2019). The RFR algorithm is an ensemble method that uses bootstrap sampling. Multiple samples are extracted from the original sample with replacement, each bootstrap sample is modeled by a decision tree, and then multiple decision trees are combined by voting to determine the final estimation (Breiman, 2001). Because multiple different decision trees are integrated, RFR is robust against overfitting (Yu et al., 2016).

The RFR models were implemented using Python version 3.7 (Google Inc., Mountain View, California, USA). The number of RFR decision trees was set to 100, the number of seeds used by the random number generator was set to 15, and the maximum depth of the decision tree was determined so that each leaf is “pure” or until all leaves contain less than the minimum number of samples needed to split the internal nodes. The other parameters used the default settings of the Python sklearn package.

To ensure a fair and comprehensive evaluation of the constructed model, we randomly selected 80% of the samples of the measured maize yields to train the model and used the remaining 20% to determine the accuracy of the yield estimates produced by this method.

### 2.6 Assessment of model accuracy

Three evaluation indicators were used to test the model accuracy: the coefficient of determination R^2^, the root mean square error (RMSE), and relative RMSE (rRMSE). These evaluation metrics are calculated as follows:


(4) $$R^{2}=1-\frac{\sum_{i=1}^{n}{\left(X_{model,i}-\bar{X_{obs}}\right)}^{2}}{\sum_{i=1}^{n}{\left(X_{obs,i}-\bar{X_{obs}}\right)}^{2}}$$

(5) $$RMSE=\sqrt{\frac{\sum_{i=1}^{n}{\left(X_{model,i}-X_{obs,i}\right)}^{2}}{n}}$$

(6) $$\text{r}RMSE=\frac{RMSE}{\bar{X_{obs}}}\times 100\%$$

where *X*_*model*,*i*_ and *X*_*obs*, *i*_ are the estimated yield and observed yield for plot *i*, respectively, $\bar{X_{obs}}$ is the average of the observed yields, and *n* is the number of samples.

## 3 Results

### 3.1 Construction of lodging index

**Figure 6:**
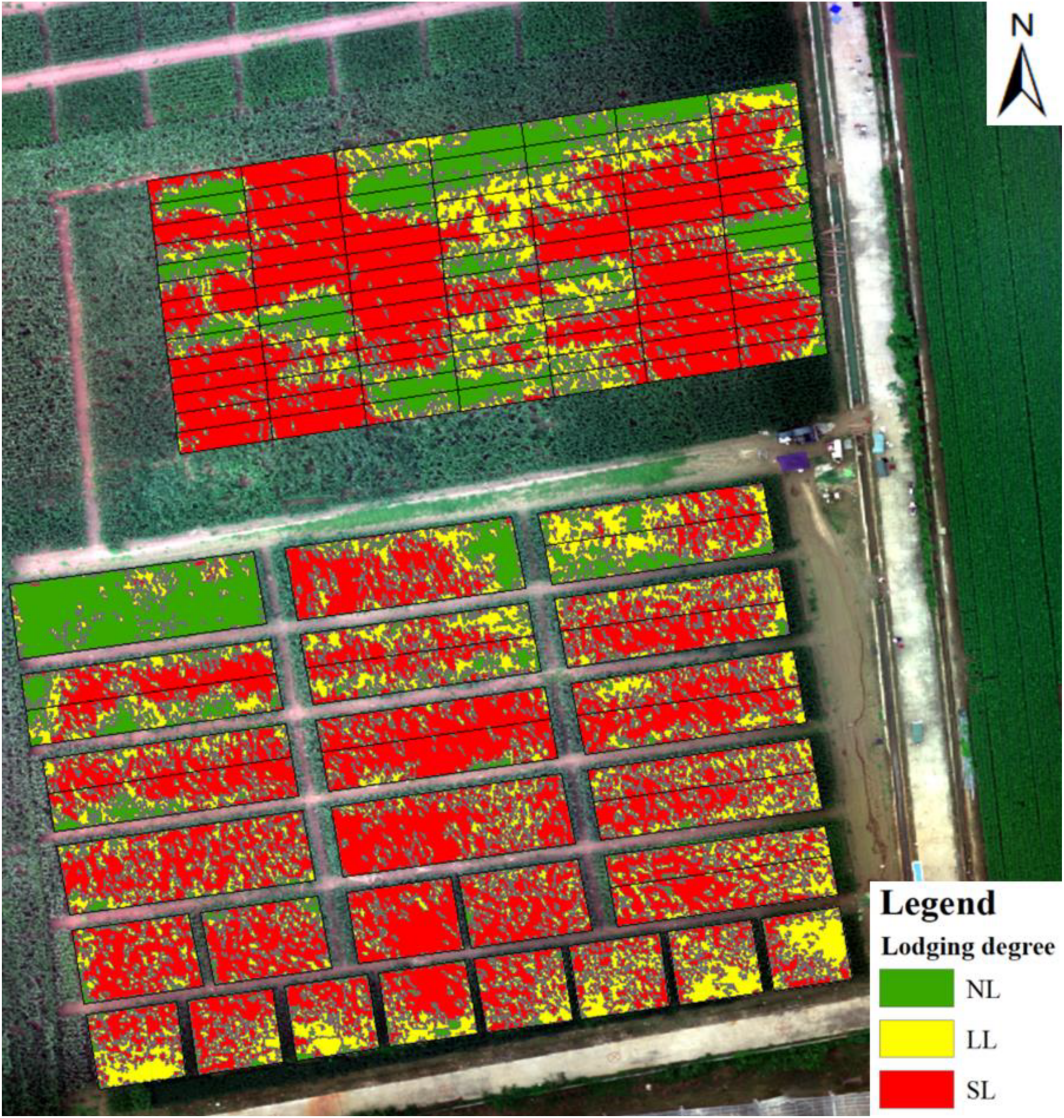
Distribution of pixel-level degree of maize lodging.

**Figure 7:**
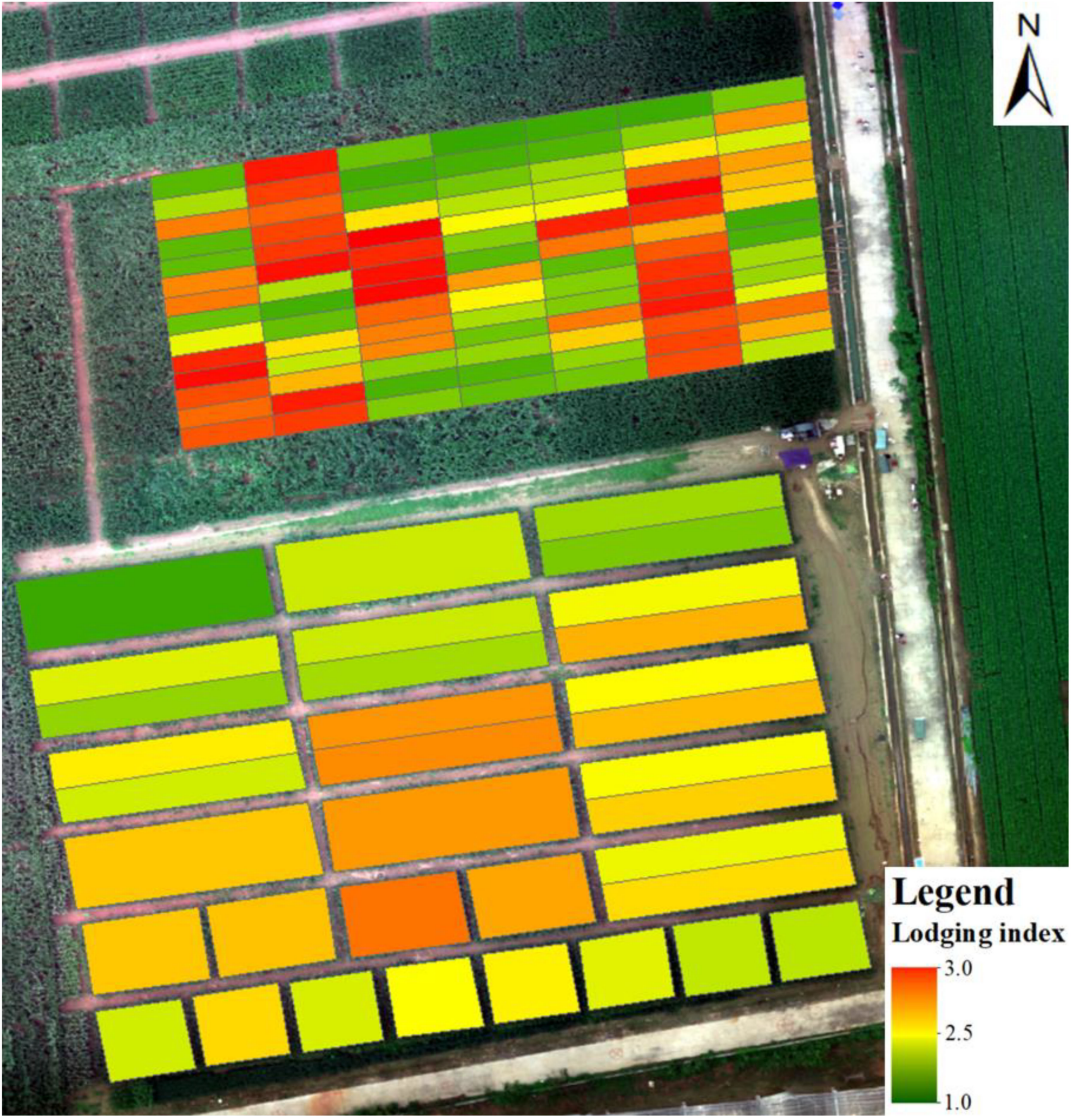
Distribution of lodging index for each plot.

The pixel-level map of lodging degree (**Figure 6**) for each test plot is derived from the pixel-size degree of lodging extracted by Wang (2021). The classification accuracy of using the random forest classifier for lodging degree at pixel size is 86.96%; for more details, see Wang (2021). The result shows that the degree of lodging differs at different positions within the same plot. Therefore, it is hard to describe the degree of lodging of the plot only by lodging area or the lodging degree at pixel size. The lodging index of each plot is shown in **Figure 7**, where a larger lodging index indicates a greater degree of lodging in the plot, and a lower degree of lodging means that the lodging degree of the plot is lighter. The lodging degree of each plot represented by the lodging index (**Figure 7**) is consistent with the spatial distribution of the pixel-level lodging degree (**Figure 6**). Thus, the lodging index is used to analyze how lodging affects yield estimates.

**Figure 8:**
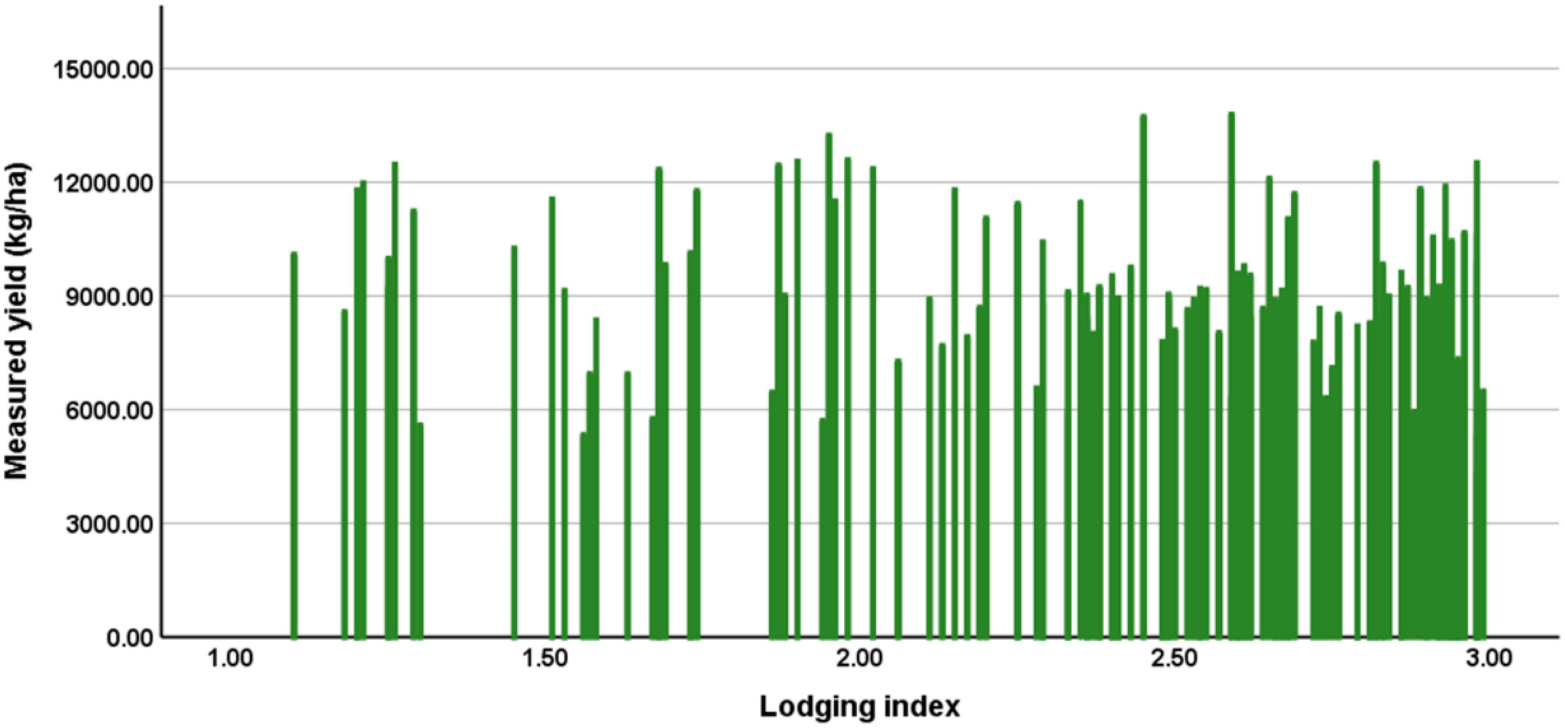
One-to-one correspondence between the lodging index and the measured yield of each plot.

To further describe the relationship between the lodging index and yield, we plot the measured yield versus the lodging index in **Figure 8**. There was no obvious increasing or decreasing trend in the measured maize yield with increasing lodging index.

### 3.2 Estimation of maize yield without lodging index

**Figure 9:**
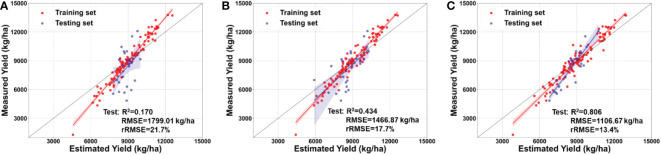
Estimation of maize yield by integrating multimodal data, but without considering the lodging index, at the **(A)** tasseling stage (VT), **(B)** milking stage (R3), and **(C)** denting stage (R5).

Differences appear in the estimated maize yield in different growth stages, and the yield-estimation model identifies the optimal harvest time. The results show that the models developed based on UAV images obtained at different growth stages perform at various levels (**Figure 9**). The model developed at the R5 stage performs best, with R^2^ = 0.806, RMSE = 1106.67 kg/ha, and rRMSE = 13.4%. The model developed at the VT stage performs the worst, with R^2^ = 0.170, RMSE = 1799.01 kg/ha, and rRMSE = 21.7%. The results indicate that models developed at later growth stages perform better than models developed at early growth stages, which is consistent with previous results (Johansen et al., 2020; Li et al., 2021).

**Figure 10:**
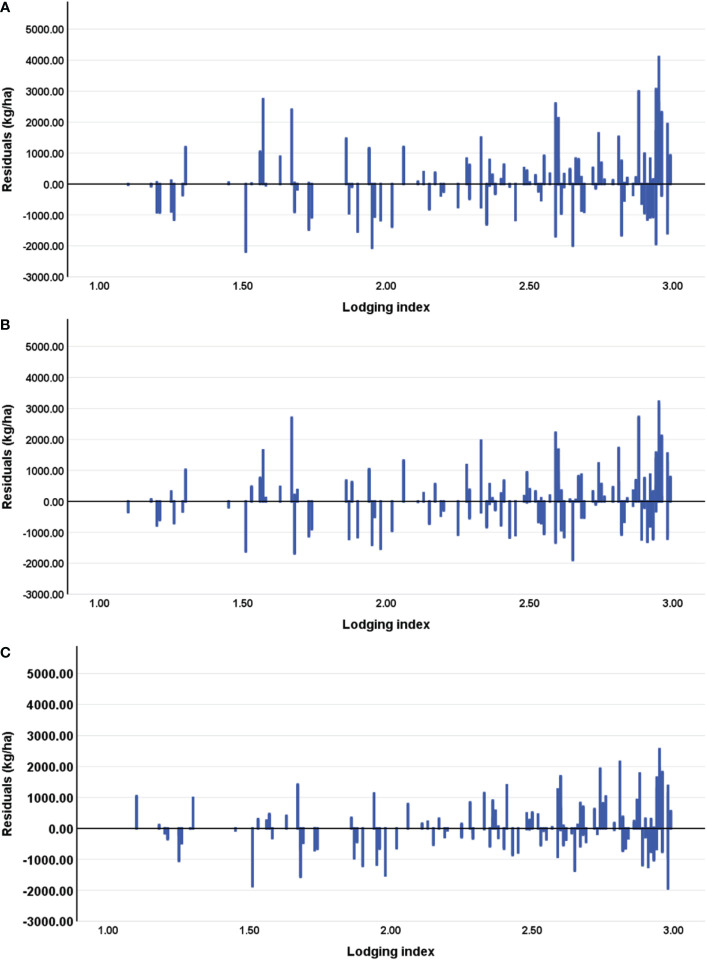
Residuals of yields estimated without considering the lodging index at the **(A)** tasseling stage (VT), **(B)** milking stage (R3), and **(C)** denting stage (R5).

To further explore whether lodging affects yield estimation, the deviation between the estimated and measured yields is plotted as a function of lodging index. As shown in **Figure 10**, no clear relationship appears between the estimation error and the lodging index. Large estimation errors appear mainly in plots with small lodging index or larger lodging index. In addition, at all three growth stages of maize, the yield of plots with a small degree of lodging tends to be underestimated with respect to the actual yield, whereas the yield of the plots with severe lodging degree is clearly overestimated. The results indicate that the yield estimates are influenced by lodging. Thus, lodging must be considered to accurately estimate yields under lodging conditions.

### 3.3 Accurate estimation of maize yield with lodging index

**Figure 11:**
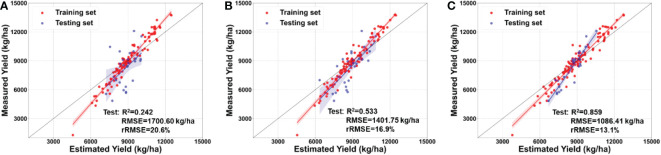
Maize yield estimated by integrating multimodal data with lodging index at the **(A)** tasseling stage (VT), **(B)** milking stage (R3), and **(C)** denting stage (R5).

**Figure 11** shows the yield estimated by integrating into the model the canopy spectral, structural, and textural information and the lodging index. For the model developed at the same growth stage, the model including the lodging index performs better than without the lodging index. For models including the lodging index, a model developed at the later growth stages performs better than a model developed at early growth stages, which performs similarly to a model without the lodging index. For a model developed at the VT stage, R^2^ increases from 0.170 to 0.242 and the RMSE decreases from 1799.01 to 1700.60 kg/ha. For a model developed at the R3 stage, R^2^ increases from 0.434 to 0.533 and the RMSE decreases from 1466.87 to 1401.75 kg/ha. For a model developed at the VT stage, R^2^ increases from 0.806 to 0.859 and the RMSE decreases from 1106.67 to 1086.41 kg/ha. These results indicate that the lodging index is a useful variable for accurately estimating maize yield when lodging occurs.

**Figure 12:**
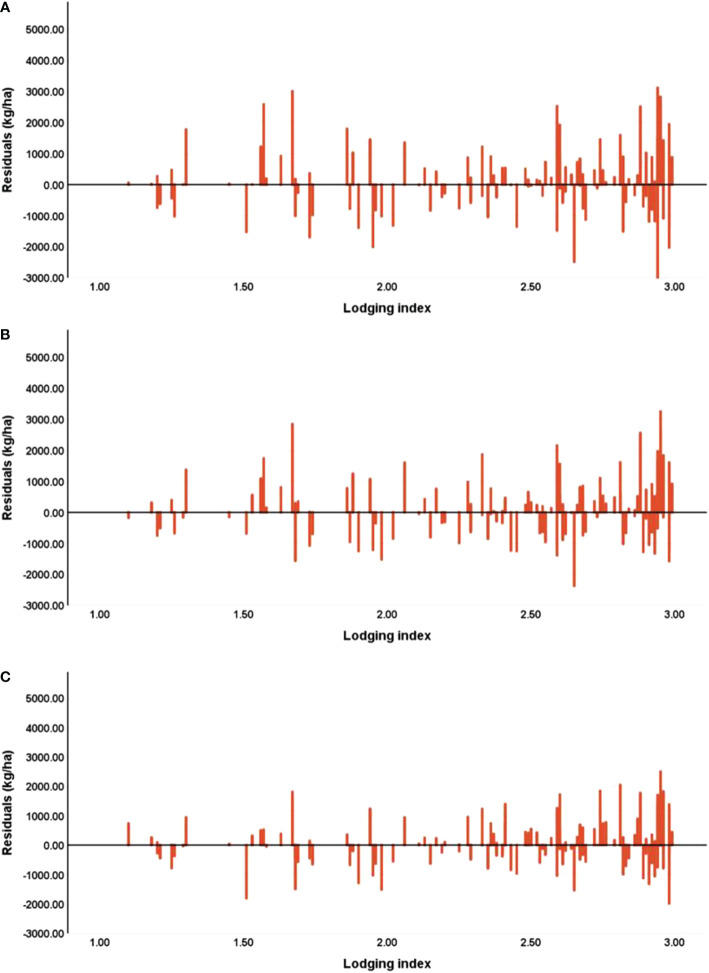
Estimation bias between measured yield and estimated yield, at the **(A)** tasseling stage (VT), **(B)** milking stage (R3), and **(C)** denting stage (R5).

To test the importance of the lodging index in yield estimation, **Figure 12** shows the relationship between the residual of the estimation model (i.e., the estimated yield minus the measured yield) and the lodging index. Upon integrating the lodging index into the maize yield estimation model for all three maize growth stages, plots with a low (high) lodging index experience more (less) overestimation but less (more) underestimation. In addition, the estimation residuals are smaller at the R5 stage than at the VT and R3 stages, although adding the lodging index. In general, adding lodging information to the model reduces the underestimation of yield in areas of slight lodging and reduces overestimation in areas of severe lodging, so the model performs better in the early stage after lodging than in the later stage.

## 4 Discussion

The construction of the lodging index should consider not only the degree of lodging but also the lodging area (Kendall et al., 2017). The lodging index proposed herein integrates the pixel-level degree of lodging and the fraction of the lodging area in the plot. The calculated lodging index well characterizes the degree of lodging of each plot (cf. **Figures 6**, **7**), but subtle differences still exist where the lodging degree is underestimated. In addition, the lodging index represents the entire plot, but omits details within plots. Future work should construct a lodging index that represents more comprehensively the lodging situation of each community.

This study uses the spectral, structural, and textural information of the maize canopy to estimate maize yields. The model developed at the R5 stage has R^2^ = 0.806, RMSE = 1106.67 kg/ha, and rRMSE = 13.4%, which is consistent with previous studies (Rischbeck et al., 2016; Feng et al., 2020; Ramadanningrum et al., 2020). Integrating the lodging index with canopy spectral, structural, and textural information improves maize-yield estimates (**Figures 8**, **10**) because lodging occurred in the field. The main reason for these results may be that the lodging index constructed herein provides useful information about the degree of lodging of each plot under natural conditions.

**Figure 13:**
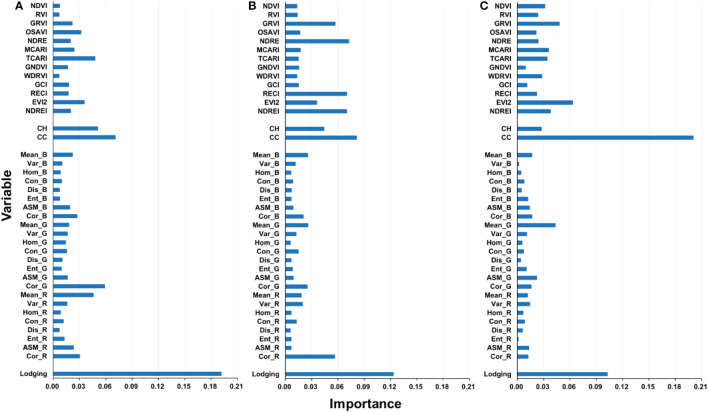
Variable importance of yield estimating model in different growth stages: **(A)** VT stage, **(B)** R3 stage, and **(C)** R5 stage.

The variable importance at different growth stages presented in **Figure 13** also shows that the CC and lodging index are important for estimating maize yields at all three growth stages. This shows that the lodging index may offer additional information associated with the growth status of maize. In addition, different variables are not screened to improve the performance of the model. Previous studies have shown that a strong linear correlation exists between vegetation indices, whereas each variable produces different effects on retrieving vegetation parameters (Zeng et al., 2022). Therefore, multiple variables must be integrated to estimate yield with better accuracy.

The improved accuracy of yield estimation in the VT and R3 stages is slightly greater than that in the R5 stage, indicating a decreased response of maize plants to lodging as they grow. This is consistent with the varying importance at different growth stages presented in **Figure 13**. The importance of the lodging index in RFR modeling decreases as the growth stage approaches maturity. Such decreases may be attributed to the natural self-recovery of maize plants and the manual measures taken. The self-recovery of maize plants could change the canopy characteristics (Hu et al., 2021), which leads to less difference between lodged and non-lodged plants. Moreover, manual measures were applied within 3 days after lodging occurred to help the maize plants return to the upright state. These measures may also reduce the influence of lodging on yields and thereby weaken the effect of the lodging index on yield estimation. In addition, the response of maize plants to lodging decreases with the growth of maize plants. In this study, all experimental plots were analyzed together to verify the stability of the model, and the role of lodging in yield estimation in a variety of experiments and nitrogen treatments was not considered. Thus, future studies should take this into consideration.

Regardless of whether the lodging index is included in the yield estimation model, the model developed in the R5 stage is the most accurate of the three growth stages, and the model developed in VT stage is the least accurate. The main reason for this result may be that pollination does not start at the VT stage, and many factors can later affect the development of seeds. For example, the canopy characteristics might change with the rapid growth of the crop after lodging occurs due to the self-recovery of maize (Han et al., 2018). As the maize plants grow closer to harvesting, the development of seeds the canopy characteristics tend to stabilize (Song et al., 2016), which would improve the correlation between the final grain yields and the canopy characteristics. Therefore, estimating crop yield at the early growth stage produces greater error than estimating crop yield at later growth stages.

## 5 Conclusion

In this study, the RGB and multispectral images obtained from a low-altitude UAV are used to estimate the grain yield of various varieties of maize with different nitrogen fertilization treatments. The canopy spectral, structural, and textural information were integrated into the RFR algorithm for estimating maize yield. In addition, to study how lodging affects yield estimation, a lodging index was developed to quantify the degree of lodging of each plot. The results lead to the following main conclusions: (1) The lodging index developed herein accurately quantifies the degree of lodging of each plot. (2) Including the lodging index into the yield-estimation model leads to more accurate crop yield estimates, and the model performs better in the early stage of maize growth than that in the later stage of maize growth. (3) The maize yield can be accurately estimated by integrating spectral, structural, textural, and structure information of the maize canopy with the lodging index, especially in the R5 stage, which gives R^2^ = 0.859, RMSE = 1086.41 kg/ha, and rRMSE = 13.1%, followed by the R3 stage, with the VT stage producing the least accurate yield estimates.

Future efforts to improve UAV-based maize-yield estimation under various lodging conditions should focus on developing a more comprehensive lodging index, exploring how lodging affects yield estimation, and seeking new ways to integrate lodging information into yield estimation.

## Data availability statement

The raw data supporting the conclusions of this article will be made available by the authors, without undue reservation.

## Author contributions

BM, JX, HY, NC, WW and HX contributed to conception and design of the study. YL and LM organized the database. YL and ZW performed the statistical analysis. YL and CN wrote the first draft of the manuscript. YL, XJ and CN wrote sections of the manuscript. All authors contributed to the article and approved the submitted version.
